# Complicated ulceroglandular tularemia

**DOI:** 10.1007/s15010-025-02691-w

**Published:** 2025-12-10

**Authors:** Dominik Trautmann, Tonio Naka, Beate Gruener

**Affiliations:** Internal Medicine III, Division for Infectious Diseases, University Hospital Ulm, Ulm, Germany

**Keywords:** Ulceroglandular tularemia, Francisella spp., Francisella tularensis, Fever of unknown origin, Lymphadenopathy

A 46 years old female physiotherapist presented herself with a painful unilateral cervical lymphadenopathy, persisting already for several weeks with a growing size to about 5 × 5 centimeters. The swelling was accompanied with repeated fever and chills. She denied traveling in recent years and her family members had no similar complaints. She looked after the horses in her own stable, including making hay and getting frequently mosquito bites. The further physical examination was unremarkable.

A calculated antibiotic treatment with an aminopenicillin, started by her primary care physician, showed no improvement so a further diagnostic work-up was performed [1, 2]. 

As the incidence of tularemia in Europe is rising clinicians should consider it a differential diagnosis in case of lymphadenopathy [3, 4, 5]. *Francisella spp.* are resistant to beta-lactams and frequent routes of infection include inhalation of aerosolized particles, arthropod bites and contact with contaminated hay or infected animals [6].

A first lymphonodal biopsy showed a granulomatous florid inflammation without signs of malignancy or mycobacteria. A taken serology tested highly positive for *Francisella*- IgM, leading to the diagnosis “ulceroglandular tularemia”. Doxycyclin was prescribed for three weeks but local swelling was worsening and continuously painful. Additionally she got ankle edemas, rated as parainfectious complication. A decompressing drainage of the abscessing lymph node demonstrated a positive Real-Time PCR for *Francisella tularensis holarctica* DNA. *Francisella*-IgM continued to be high in the serology. A combination therapy with gentamicin for 7 days, ciprofloxacin for 14 days plus an prednisolone treatment were given after which her symptoms completely resolved [1, 2].

**Fig. 1:**
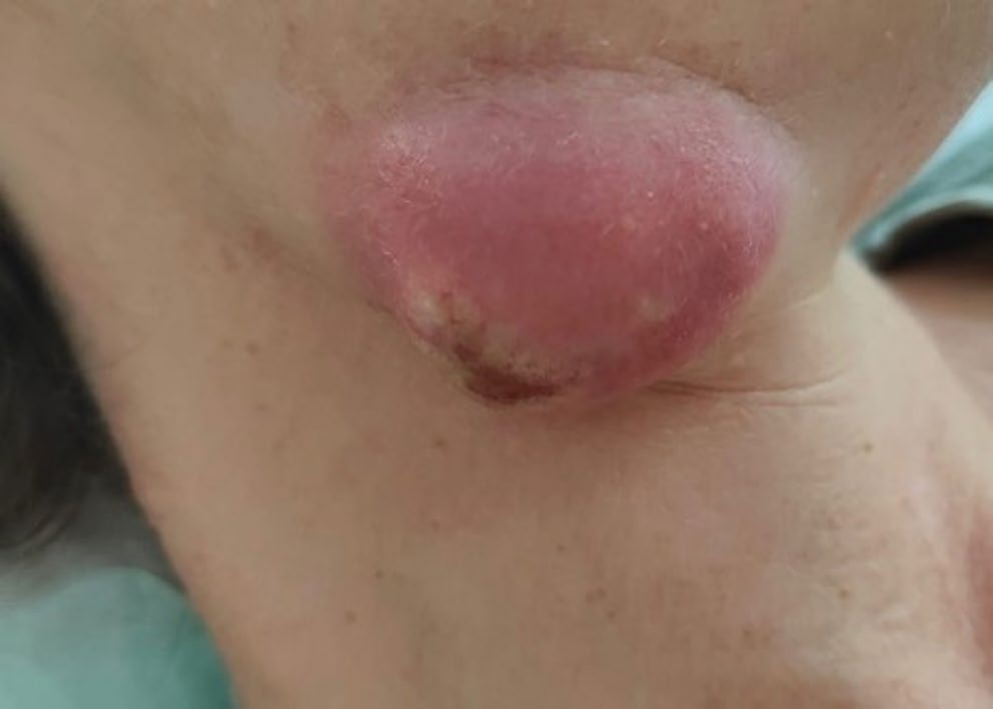
The patient’s neck showing a painful und red swelling, located in the right submandibular area (diameter of 5 cm)

**Fig. 2:**
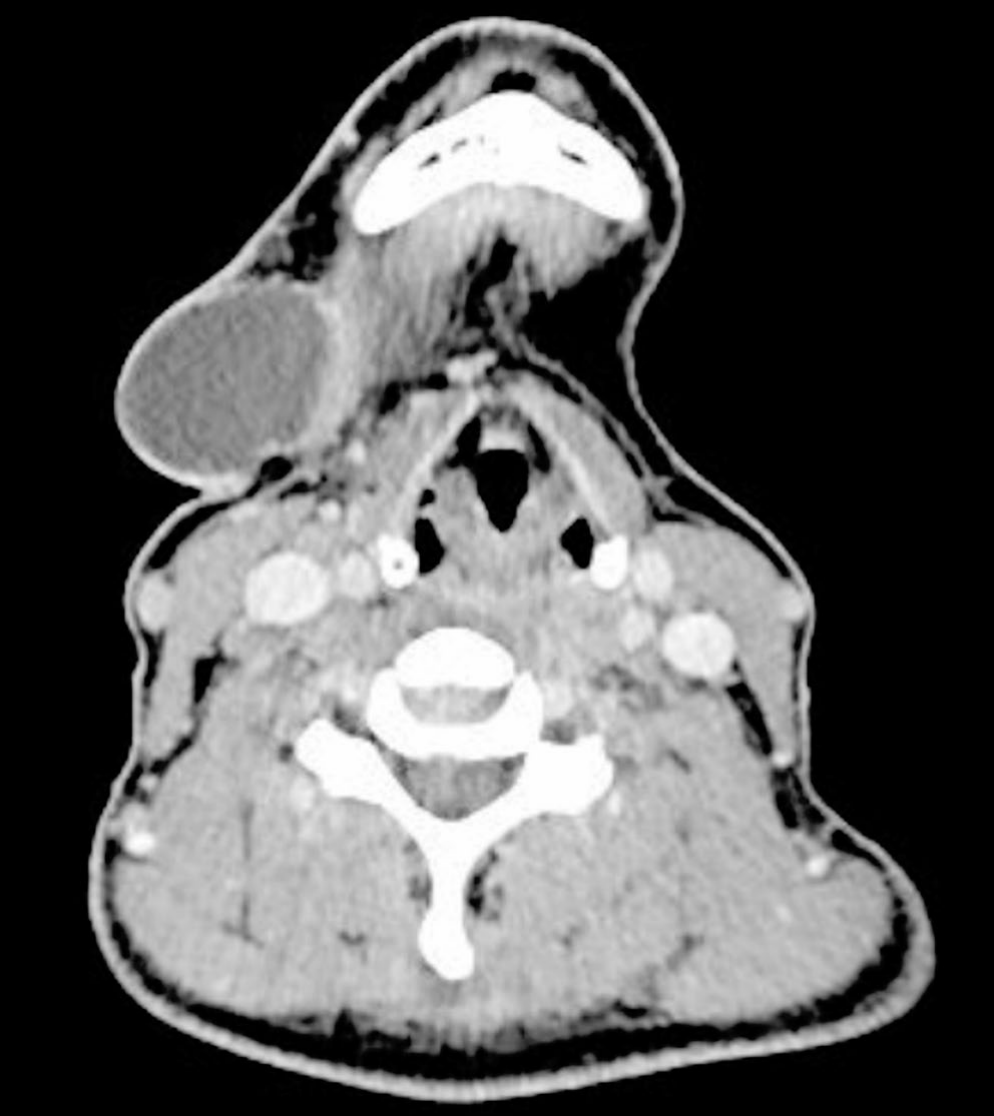
Computer tomography of the neck showing a large lymphonodal swelling in the right submandibular area

## Data Availability

No datasets were generated or analysed during the current study.
